# Electrical and Self-Sensing Properties of Ultra-High-Performance Fiber-Reinforced Concrete with Carbon Nanotubes

**DOI:** 10.3390/s17112481

**Published:** 2017-10-29

**Authors:** Ilhwan You, Doo-Yeol Yoo, Soonho Kim, Min-Jae Kim, Goangseup Zi

**Affiliations:** 1 School of Civil, Environmental and Architectural Engineering, Korea University, 145 Anam-ro, Seongbuk-gu, Seoul 02841, Korea; ih-you@korea.ac.kr (I.Y.); g-zi@korea.ac.kr (G.Z.); 2 Department of Architectural Engineering, Hanyang University, 222 Wangsimni-ro, Seongdong-gu, Seoul 04763, Korea; tnsgh0905@hanyang.ac.kr (S.K.); mandufa345@naver.com (M.-J.K.)

**Keywords:** ultra-high-performance fiber-reinforced concrete, steel fiber, carbon nanotube, electrical property, self-sensing capacity

## Abstract

This study examined the electrical and self-sensing capacities of ultra-high-performance fiber-reinforced concrete (UHPFRC) with and without carbon nanotubes (CNTs). For this, the effects of steel fiber content, orientation, and pore water content on the electrical and piezoresistive properties of UHPFRC without CNTs were first evaluated. Then, the effect of CNT content on the self-sensing capacities of UHPFRC under compression and flexure was investigated. Test results indicated that higher steel fiber content, better fiber orientation, and higher amount of pore water led to higher electrical conductivity of UHPFRC. The effects of fiber orientation and drying condition on the electrical conductivity became minor as sufficiently high amount of steel fibers, 3% by volume, was added. Including only steel fibers did not impart UHPFRC with piezoresistive properties. Addition of CNTs substantially improved the electrical conductivity of UHPFRC. Under compression, UHPFRC with a CNT content of 0.3% or greater had a self-sensing ability that was activated by the formation of cracks, and better sensing capacity was achieved by including greater amount of CNTs. Furthermore, the pre-peak flexural behavior of UHPFRC was precisely simulated with a fractional change in resistivity when 0.3% CNTs were incorporated. The pre-cracking self-sensing capacity of UHPFRC with CNTs was more effective under tensile stress state than under compressive stress state.

## 1. Introduction

Civil structures are frequently constructed using concrete due to its several advantages including low price, high compressive strength, and good durability. However, in general, the structural integrity of reinforced concrete structures continuously declines with age, arising from concrete creep or shrinkage, unintended external loads, corrosion of internal steel reinforcements, freeze–thaw damage, etc. The deterioration of old reinforced concrete structures can lead to their sudden collapse, causing numerous casualties, as has been witnessed several times worldwide. Thus, it is very important to accurately monitor the structural integrity of existing structures based on a structural health monitoring (SHM) technique. Use of the SHM technique allows structural integrity monitoring of large-scale structures based on real-time data, so that engineers can efficiently repair them if necessary.

SHM has been carried out using various types of strain gauge sensors [1,2,3], which in most cases are embedded in or attached to a structure. However, they have several drawbacks that limit their practical application. First, there is uncertainty regarding the properties of the bond between the surface of a structure and the sensor attached to it. Second, the SHM technique using conventional sensors is difficult to scale up to large-scale structures due to management issues. Lastly, it is necessary to accurately predict the weakest zone where failure is expected to occur, for which the sensor needs to be located nearby, because conventional sensors are small and discontinuous. To overcome these limitations, several researchers [4,5,6,7,8,9,10,11,12,13,14,15,16,17] have worked to develop self-sensing cement composites with conductive materials based on the piezoresistive properties.

To induce self-sensing properties of cement composites the conductivity should be secured by incorporating conductive materials such as carbon-based materials, copper and nickel powders, because plain cement having the resistivity more than 10,000 Ω·cm [4] is considered to be similar with insulators. The conductive materials provide conductive pathways for cement composites through direct contact with each conductive materials and tunneling effects that can be generated as distance between each conductive materials is quite close within roughly less than 10 nm [18]. The cement composites incorporating conductive materials with effective amount, called percolation threshold, that the resistivity of cement composites no longer decrease, can reduce the resistivity similar to level of semiconductors (1–100 Ω·cm). In addition, when the cement composites with conductive materials are subjected to external load, their geometries are deformed and their resistivity is also changed. This is due to change in resistance of conductive pathways in cement composites because, based on the piezoresistive effect, deformed geometries of conductors can affect current flow. Consequently, with similar principle of typical strain gauge sensors, it is possible to evaluate self-sensing properties of cement composites by measuring change in resistance.

Banthia et al. [12] examined the electrical resistivity of cement paste including micro steel and carbon fibers and reported that the resistivity of plain paste was notably reduced by adding the fibers, and that adding carbon fibers was more effective in improving the paste’s conductivity than adding steel fibers, inconsistent with the findings of Wen and Chung [13]. In response to this inconsistency, Yoo et al. [15] insisted that the conductivity of cement composites is more closely related to fiber connectivity than the type of fibers, i.e., steel and carbon fibers. Azhari and Banthia [14] also noted that, compared to the use of carbon fibers only, the combined use of carbon fibers and carbon nanotubes (CNTs) provides better quality signal, increased sensor sensitivity, and improved reliability under compressive loads of arbitrary loading rate. Yoo et al. [4] studied the effects of carbon-based nanomaterial additives, namely CNTs, graphene, and graphite nanofibers, on electrical and piezoresistive properties of cement paste. Based on numerous test results, they reported that CNTs yielded the best piezoresistive performance, with insignificant noise, followed by graphite nanofibers and graphene, given the constant additive volume fraction of 1%. Han et al. [11,16] conducted intensive studies to develop conductive cement-based composites with CNTs, which can simulate cyclic behaviors of compressive stress based on changes in the electrical resistance. They were able to apply these CNT/cement composites for traffic monitoring [11]. Several other researchers [7,9] investigated the percolation threshold of CNTs in cement composites. D’Alessandro et al. [9] suggested a percolation threshold of CNTs around 1% by cement weight, much higher than that of 0.3–0.6% by cement weight proposed by Kim et al. [7] and the optimum value proposed by Han et al. [17]. This dissimilarity might be attributed to differences in the mixture proportions, CNT properties, and the inclusion or omission of a sonication treatment. Likewise, numerous studies have been performed to develop conductive and self-sensing cement-based materials.

However, because most of the previous studies have been performed using ordinary cement paste or mortar, their results do not apply to special types of concretes such as ultra-high-performance fiber-reinforced concrete (UHPFRC), which exhibits totally different mechanical properties. UHPFRC, which was first introduced in the mid-1990s, has attracted much attention from engineers in recent years due to its several advantages such as excellent strength (compressive strength of at least 150 MPa and tensile strength of at least 8 MPa), ductility, durability, and fatigue resistance. Such excellent mechanical properties can be achieved by using the quite low water-to-binder (W/B) ratio of about 0.2 and including high-fineness admixtures and a high volume of micro steel fibers. Unfortunately, only very limited studies have been published on development of conductive UHPFRC materials, so it is still unclear whether or not UHPFRC itself is sufficient for self-sensing monitoring, and if not, what kinds of supplementary materials are required for preparation of conductive UHPFRC. Dong et al. [19] improved the electrical conductivity of UHPFRC and imparted it with self-sensing ability by using short-cut super-fine stainless wire (SSSW). Including 0.5% SSSW reduced the electrical resistivity by up to four orders of magnitude, and using SSSW of 20 μm wire diameter was more effective than that of 8 μm wire diameter. However, even though they achieved sufficient compressive strength in this way, the replacement of steel fibers by SSSW decreased the flexural strength considerably (approximately below 50%) compared to that of conventional UHPFRC [20]. Therefore, there is a continuing need to develop a conductive self-sensing UHPFRC material without any significant deterioration in mechanical properties for use in a wide range of SHM applications.

Accordingly, in the present work, we developed a new type of conductive UHPFRC material using both steel fibers and CNTs. The specific objectives of this study included evaluating: (1) the effects of steel fiber content, orientation, and pore water content in hardened paste on electrical and piezoresistive properties of UHPFRC under compression; (2) the effects of a sonication process on the electrical conductivity of UHPFRC with CNTs; and (3) the effects of CNT content on mechanical strength and self-sensing capacities of UHPFRC under both compression and flexure.

## 2. Experimental Program

### 2.1. Constituents, Mix Proportions, and Specimen Preparation

Table 1 lists the mixture proportions used in this study. As cementitious materials, Type I Portland cement and zirconium silica fume (Zr SF) were used; Table 2 lists their chemical compositions and physical properties. Fine silica sand of particle diameter 0.2–0.3 mm was used as fine aggregate; coarse aggregate was excluded from the mixtures. The inclusion of coarse aggregate imparts several advantages such as lower production cost and shorter mixing time, while not distinctively reducing compressive strength [21]. However, it also decreases tensile or flexural strength by reducing the bond strength between the fiber and the matrix, mainly caused by decreased amount of shrinkage [22]. Because the excellent tensile or flexural performance of UHPFRC is a main reason for using this material, and because it is already much more expensive to produce than ordinary concrete, coarse aggregate was not used in the present work. Very fine silica flour of median particle size 4.2 μm was included as a filler to improve the homogeneity by the filling effect, and the amounts and particle sizes of silica sand and flour were determined based on a packing density theory [23]. A W/B ratio of 0.2 was adopted. Because this UHPFRC mixture had a low W/B ratio and large amounts of fine admixtures, its viscosity was quite high, leading to very sticky mixtures. Thus, to ensure sufficient fluidity, a high-range water reducing agent, a polycarboxylate superplasticizer (SP) with a density of 1.01 g/cm^3^ and dark brown color, was additionally incorporated. The fluidity of the mixture was evaluated based on a flow table test according to ASTM C1437 [24]. A detailed mixing sequence for UHPFRC can be found in a previous study [25].

**Figure 1:**
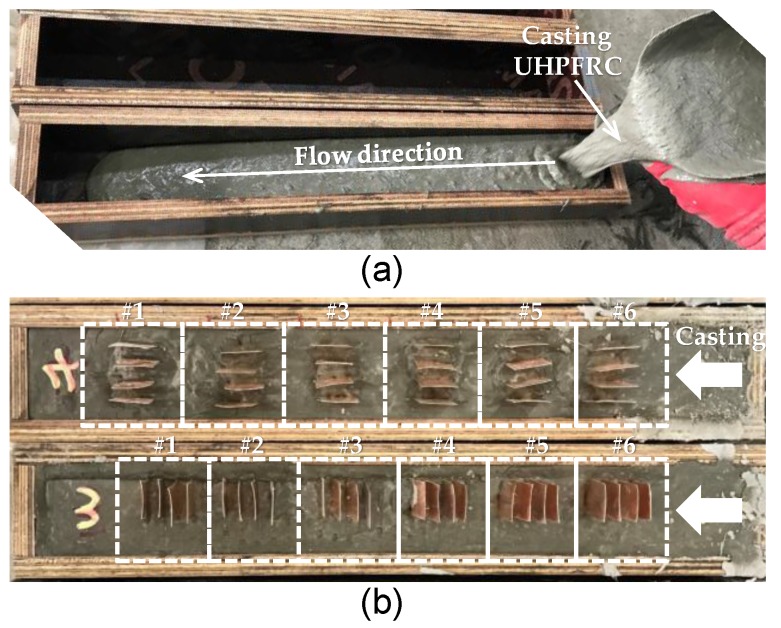
Fabrication process of UHPFRC specimens: (**a**) casting method; and (**b**) direction of copper plates according to casting direction.

In Part I of this study, to investigate the effects of fiber volume content, orientation, and desiccation of pore water on the electrical conductivity of UHPFRC, three different volume fractions (*v_f_*) of straight steel fibers were adopted: 1%, 2%, and 3%. Because UHPFRC exhibits very high bond strength, to prevent fiber fracture before complete pullout, high-strength straight steel fiber was adopted. Table 3 summarizes the geometrical and physical properties of the steel fiber used. UHPFRC is a type of self-consolidating concrete, so samples or elements can be fabricated by casting it at certain points and allowing it to flow. The fiber orientation of UHPFRC varies according to the casting method because of different gradients of flow velocity. In particular, for shear flow, mostly found in beam elements, fibers tend to align in the direction of flow due to the torque caused by wall friction, leading to a parabolic flow gradient [20]. In this case, many fibers are aligned in the specimen’s longitudinal direction, thereby improving the post-cracking tensile and flexural performance. The fiber orientation affects the electrical properties in addition to the mechanical properties by affecting the conductive pathways that form within the cement; these pathways have the most significant influence on the electrical conductivity of cement composites that include carbon-based nanomaterials and fibers [5,6]. Thus, thin UHPFRC beams of cross-sectional area of 50 × 50 mm^2^ and length 400 of mm were fabricated by casting at the end of the beam and allowing the concrete to flow to the other end (Figure 1a). Then, copper plates were inserted into a fresh UHPFRC mixture in the parallel and vertical directions of flow, as illustrated in Figure 1b. The movement of current in the specimen with copper plates in the direction parallel to concrete flow will be perpendicular to the direction of fiber alignment, whereas the current movement in the specimen with copper plates in the direction perpendicular to flow will be parallel to the direction of fiber alignment. Thus, these insertions allowed examination of the effects of fiber orientation on the electrical properties of the UHPFRC. In addition, to evaluate the effects of fiber orientation and content on the electrical resistivity and piezoresistivity of UHPFRC, the thin beam was cut into eight pieces with a dimension of 50 × 50 × 50 mm^3^ using a diamond blade. The pieces collected from the ends of each beam were not used in the tests because their fiber orientations might have been disturbed and not well aligned. The fibers in the cube formed closest to the casting point were probably more randomly oriented than others due to their short flow distance, whereas the fibers in the cube at the opposite end were likely aligned more perpendicular to the direction of flow than others due to the end wall effect.

In Part II, various amounts of CNTs, namely 0.1%, 0.3%, and 0.5% (by volume), were incorporated into UHPFRC mixtures including 2 vol % straight steel fibers to impart self-sensing ability. The UHPFRC mixture was designed to contain 2 vol % steel fibers, because such mixtures are commercially available in North America [26]. Therefore, the main purpose of the Part II is to investigate the effect of adding CNTs on the self-sensing ability of commercial UHPFRCs. Table 4 lists the geometrical and physical properties of the CNTs used in this study. To develop a mixture for casting self-sensing UHPFRC, various cubic specimens of cross section of 50 × 50 mm^2^ and height of 50 mm and prismatic beams of dimensions of 100 × 100 × 400 mm^3^ were fabricated and tested.

First, to examine the effect of sonication, 12 cubic specimens made of UHPFRC mixtures including 0.5 vol % CNTs were prepared, six each with or without the application of a sonication process. To prepare the sonicated mixture, a Q500 Sonicator with an operating frequency of 20 kHz was used to disperse the CNTs in the mixing water. On the other hand, to prepare the unsonicated mixture, the CNTs were dispersed carefully by hand into the dry components, namely cement, Zr SF, silica sand, and silica flour, and were then premixed for 10 min before adding the mixing water. Three of the six specimens made under each condition were used to measure the compressive strength, allowing indirect evaluation of the dispersion of CNTs, and the other three were used to evaluate the electrical resistivity and piezoresistive capacity. Average values are reported from three specimens.

Second, to investigate the effect of CNT content on the electrical and self-sensing capacities of UHPFRC, three different volume fractions of CNTs were adopted, namely 0.1%, 0.3%, and 0.5%. The choice of these amounts was informed by a previous report that the percolation threshold value of CNTs for cement composites is between 0.3 and 0.6 wt %. It was obvious that the flowability of fresh UHPFRC mixture decreases with increasing content of added CNTs, based on a flow table test conducted according to ASTM C1437 [24]. Therefore, slightly higher amounts of SP, of about 20% and 50%, were added to the mixtures containing 0.3% and 0.5% CNTs, respectively. In addition, for this investigation, numerous cubes and prisms were used and tested under compression and flexure.

For both Parts I and II, the UHPFRC specimens were cured in the air for the first 48 h, after which the molds were removed from the specimens, and steam curing with heat (90 °C) was applied for 72 h to promote strength development. Then, some of the specimens were cured in the atmosphere until testing, whereas the rest were cured in a drying machine to remove residual pore water from the hardened cement matrix.

### 2.2. Test Setup for Electrical Resistance

**Figure 2:**
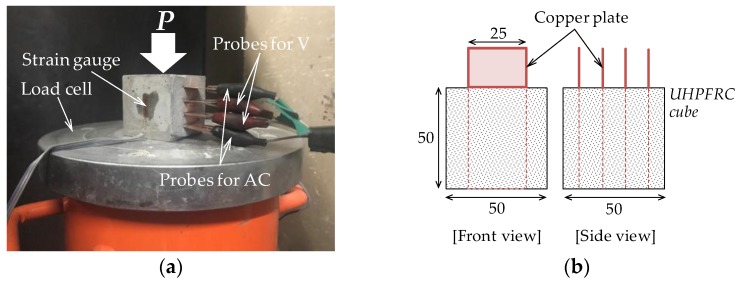
Test setup for compressive test: (**a**) test picture; and (**b**) geometrical details of cube.

To evaluate the electrical conductivity of UHPFRCs with and without CNTs, cubic specimens of dimensions 50 × 50 × 50 mm^3^ were used. An LCR multi-meter (GW Instek LCR-819) that can measure electrical properties of semiconductors, such as inductance, capacitance, and resistance, by using probe methods was adopted, as shown in Figure 2. Two outer and inner probes were connected to probes for current flows and voltage measurement, respectively. Measured changes in resistance are automatically recorded in the computer linked with LCR multi-meter. Chen et al. [28] reported that the resistance of cement composites increases with time due to a polarization effect, when a direct current method is adopted. Applying electrical current through cement paste leads to liberation of hydrogen and oxygen, forming a thin film between the electrodes and the cement paste that increases the measured resistance. Therefore, an alternative current method was used in this study, similar to that used by Banthia et al. [12] and Chen et al. [28]. Using the LCR meter, alternating current of frequency 100 kHz was applied.

### 2.3. Test Setup for Evaluating Piezoresistive and Self-Sensing Capacities

To evaluate the piezoresistive and self-sensing capacities of UHPFRC under compressive loads, a test setup was prepared as shown in Figure 2 using cubic specimens. A four-probe method was adopted. Four copper plates with a width of 25 mm were inserted into the cubic specimens completely, and their distance was 10 mm equally. Rubber pads were used as insulators between the specimen surface and the loading plates to avoid current flow into the universal testing machine (model MTS 815) through the steel fibers or CNTs. Two foil strain gauges were attached to the surface of each side of the specimen in order to measure strain in the direction of load, and their data were acquired by an independent computer linked with a data logger (TDS-303). The load was controlled by displacement with the loading rate of 0.01 mm/s. The resistance change in response to the compressive load was measured by another computer equipped with an LCR meter. Outside and inside probes were linked with the current and voltage terminals of the LCR meter, respectively.

**Figure 3:**
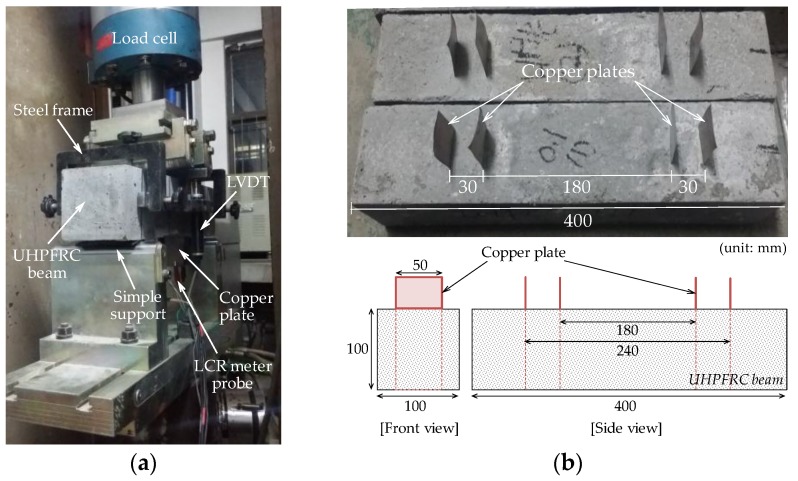
Test setup for four-point bending test: (**a**) test picture; and (**b**) geometrical details of beam.

Figure 3 shows the test setup used for measurement of the self-sensing capacities of UHPFRC in response to flexure. The well known four-point bending test method, ASTM C1609 [29], was employed to investigate the flexural performance of UHPFRC including CNTs. Four electrodes were inserted in a manner avoiding the maximum moment section, as shown in Figure 3b. They were inserted into the specimen in order to monitor flexural behavior. The beams were rotated 180° from the casting surface, and, thus, the casting surface became the bottom surface when four-point bending tests were carried out, as shown in Figure 3a. In the flexural tests, rubber pads were used at the connection between specimen and loading line. For measuring the pure deflection of specimens excluding support settlement, two LVDTs were installed vertically at the middle of the specimen using a steel frame. A foil strain gauge for measuring bending tensile strain was also attached to the middle of the bottom surface of each specimen. The uniaxial load was monotonically applied to the specimens through displacement control with a rate of 0.1 mm/min, and the load was measured from a load cell affixed to the test machine.

## 3. Experimental Results and Discussion

### 3.1. Part I: Effects of Steel Fiber Amount, Orientation, and Desiccation on Electrical Properties of UHPFRC

#### 3.1.1. Effects of Fiber Content, Orientation, and Desiccation on Electrical Resistivity

**Figure 4:**
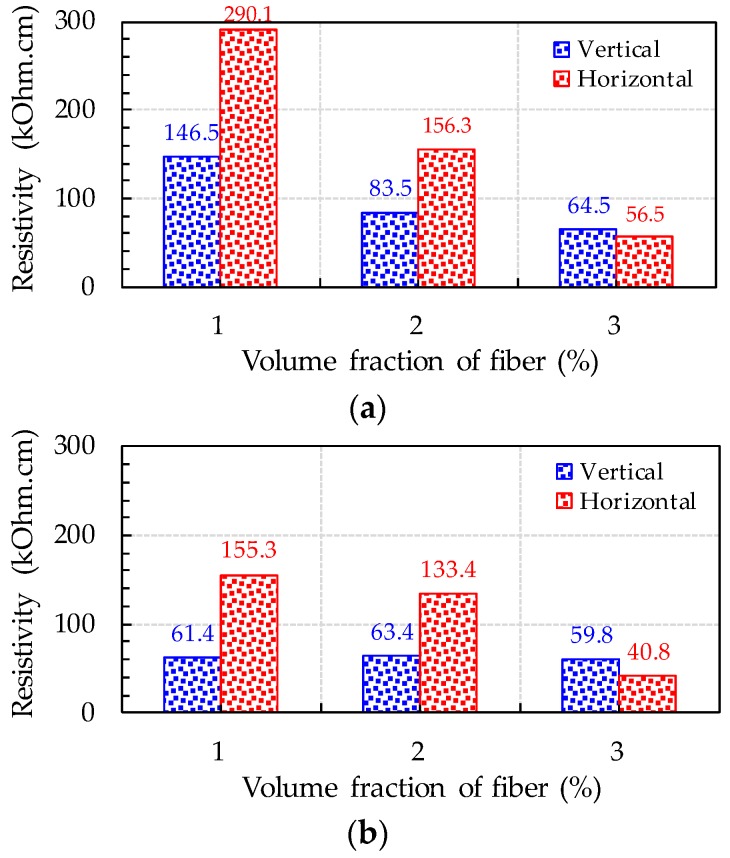
Effects of fiber volume fraction and orientation on the resistivity of UHPFRCs: (**a**) dried sample; and (**b**) ambient-cured sample (Note: Vertical, copper plates vertical to casting direction; and Horizontal, copper plates horizontal to casting direction).

Because the UHPFRC incorporated high volume contents of steel fibers, it can be expected that electrical current would be transferred though it by means of conductive pathways formed by contacts between steel fibers or electrical tunnels. Thus, the electrical conductivity of UHPFRC can be influenced by its steel fiber content and fiber orientation. In addition, electrical conductivity can be affected by the water content in the capillary pores of hardened cement paste, because if these pores are saturated, current can be more effectively transferred through the pore water [4]. Thus, the effects of steel fiber content, orientation, and desiccation on the electrical resistivity of UHPFRC are presented in Figure 4. The electrical resistivity of cubic specimen was calculated using the equation *ρ* = *R* × *A*/*l*, where *ρ* is the electrical resistivity, *R* is the resistance, *A* is the area of UHPFRC in contact with the copper electrode, and *l* is the space between the two voltage poles. The electrical resistivity of UHPFRC was significantly influenced by the fiber content, orientation, and desiccation, causing different amounts of pore water, as was expected. It was obvious that decreasing the resistivity (i.e., increasing the conductivity) of UHPFRC could be carried out by increasing the steel fiber content. This is because increasing the steel fiber content leads to the formation of more contacts between fibers and more electrical tunnels. Current is only transferred when the tunneling gap is small enough [6]. Thus, because high volume fractions of steel fibers can reduce the distances between fibers, the higher fiber volume fractions could improve the electrical conductivity. In addition, specimens with copper plates oriented perpendicular to the fiber alignment direction had lower resistivities than those with copper plates oriented in a parallel direction to the fiber alignment. The differences between the resistivities of UHPFRCs with different fiber orientations (parallel vs. perpendicular) became more significant when a lower fiber volume fraction was used. For instance, the highest electrical resistivity was found for dried horizontal sample with 1% steel fibers and (*ρ* = 290.1 kΩ·cm), 98% higher than that of dried vertical sample with identical amount of steel fibers. On the other hand, dried horizontal and vertical samples with 3% steel fibers had similar electrical resistivities, meaning that, at the fiber volume fraction of 3%, the electrical resistivity of UHPFRC was not affected by the casting method (or fiber orientation). This is because, under conditions of greater fiber concentration, the fiber alignment in the direction of concrete flow is disturbed by fiber–fiber interactions [20,30], and thus, the fibers become more randomly oriented. When UHPFRC is cast at the end of a thin beam and allowed to flow, the fibers tend to align in the flow direction due to a gradient of flow velocity arising from the wall effect [20]. Thus, such specimens are expected to have fibers aligned in the direction of concrete flow. However, increasing the amount of steel fibers disturbs fiber rotation toward the flow direction under the gradient of flow velocity, causing fewer fibers to be aligned in the flow direction. Therefore, the effect of casting method on the electrical resistivity of UHPFRC became insignificant for the high fiber volume fraction of 3%.

As shown in Figure 4, specimens cured in the air provided lower resistivity than those cured in the desiccator, regardless of the fiber content or orientation. This is because the air-cured specimens had more pore water than those dried in the desiccator, yielding higher electrical conductivity. The pore water helped to transfer the electrical current among steel fibers not in contact with each other. Interestingly, the differences in electrical resistivity among UHPFRC specimens according to drying conditions (ambient vs. desiccator) became minor with increasing volume fractions of steel fibers. For instance, the electrical resistivity of dried vertical sample with 3% fibers was found to be 64.5 kΩ·cm, which was just 8% higher than that of ambient-cured vertical sample with 3% fibers. This indicates that the effect of drying condition on the electrical resistivity of UHPFRC is mitigated by increasing the fiber volume fraction, and, at the high fiber volume fraction of 3%, similar resistivities were obtained for specimens cured either way. In summary, the electrical resistivity of UHPFRC is insignificantly influenced by fiber orientation and drying condition when the fiber volume fraction is equal to or greater than 3%. Therefore, in the following section, the piezoresistive properties of UHPFRC with 3 vol % straight steel fibers of aspect ratio (*l_f_*/*d_f_*) of 65 (13 mm/0.2 mm) were investigated.

#### 3.1.2. Piezoresistive Properties of UHPFRC with 3 vol % Steel Fibers

**Figure 5:**
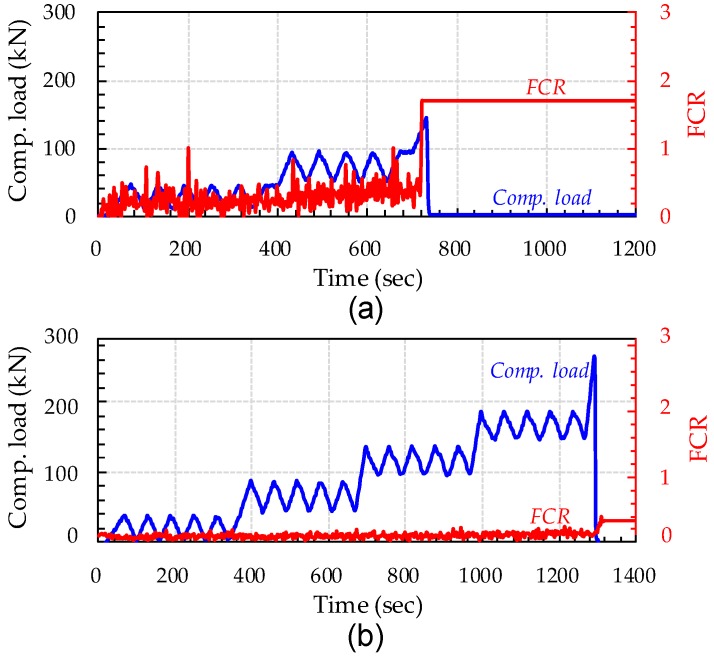
Comparative load–time and FCR–time curves of UHPFRC with 3% steel fibers: (**a**) V-series; and (**b**) H-series (Note: V, copper plates vertical to casting direction; and H, copper plates horizontal to casting direction).

Figure 5 compares curves of compressive load and fractional change in resistivity (FCR) versus time for UHPFRC with 3 vol % steel fibers, which exhibited the highest conductivity in the tests described above. Herein, FCR values were calculated according to the following equation: Δ*R*/*R*_0_ = (*R_x_* − *R*_0_)/*R*_0_, where *R*_0_ is the initial resistance of a composites, and *R_x_* is the resistance of the composite during loading. Under a compression, the FCR becomes negative because the resistivity is reduced with an increase in the load. Thus, to directly compare the FCR and the compressive load (or stress), −1 was multiplied for the measured FCR of UHPFRC cubes under the compression. To evaluate the piezoresistive properties of UHPFRC with steel fibers, cyclic compressive loading was applied. As shown in Figure 5, although the most conductive UHPFRC mixture was used, the measured FCR did not noticeably change in response to the cyclic variations in compressive load. This is inconsistent with findings by Chung [8]. Chung [8] reported that the trend of FCR well followed the trend of cyclic compressive load of cement composites including 0.72 vol % micro steel fibers with a diameter of 8 μm, although carbon fibers were more effective than steel fibers. This is because the fibers moved closer together under compression, improving their connectivity. Therefore, under compression, the FCR was negative. Interestingly, UHPFRC specimens with 3% steel fibers did not show any significant piezoresistive properties, compared to previously reported cement composites [8], as shown in Figure 5. Some possible reasons for this observation are as follows. (1) The size of steel fibers with a diameter of 0.2 mm used were too high to effectively form continuous conductive pathways. (2) Insufficient pore water in the hardened cement paste decreased the effectiveness of transferring current through the pore water. (3) Very densified microstructures of UHPFRC disturbed the connections between steel fibers, producing conductive pathways under compressive load. As shown in Figure 5, the FCR suddenly increased when the cubic specimen failed under compression. This is attributed to the fact that, because the distances between steel fibers suddenly and greatly increased when the specimen failed due to the crack localization phenomenon, the resistance at this point also steeply increased. Therefore, it is concluded that the UHPFRC including only the high volume fraction, 3%, of steel fibers is improper for use in sensing of piezoresistive properties, even though it can predict severe damage, namely compressive failure.

### 3.2. Part II: Electrical and Self-Sensing Capacities of UHPFRC with CNTs

#### 3.2.1. Sonication Effect

To evaluate the effect of sonication on the electrical properties of UHPFRC containing both steel fibers and CNTs, electrical resistivity was measured after a completion of steam curing with heat. The electrical resistivity of UHPFRC was found to be substantially reduced by including 0.5 vol % CNTs, as given in Table 5. The electrical resistivity of UHPFRC including 2 vol % steel fibers only was found to be 260.4 kΩ·cm, but the much smaller resistivities of 323 and 393 Ω·cm were obtained by including 0.5 vol % CNTs, with and without sonication, respectively. Drawbacks of sonication have been reported previously [9,31]: (1) use of a sonication process is one of the biggest limitations in practical application of SHM for large-scale structures [9]; and (2) CNTs can be damaged or broken under high sonication energy [31]. Thus, Mendoza et al. [31] insisted that, before applying the sonication process, an optimal sonication energy should be proposed that can balance the tradeoff between the degree of dispersion imparted and the resulting damage to the CNTs. Furthermore, because sonication had a minor effect of reducing the electrical resistivity of UHPFRC in this study, the sonication process was not applied in the work described in the following section to investigate the implication of CNT content on the electrical and self-sensing capacities of UHPFRC under both compression and flexure.

#### 3.2.2. Effects of CNT Content on Flowability and Compressive Behavior of UHPFRC

**Figure 6:**
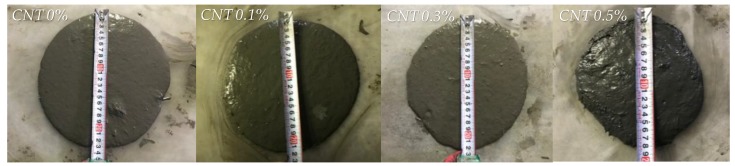
Flow table test results.

Figure 6 shows the flow values measured from flow table tests for all UHPFRC mixtures with CNTs as per ASTM C1437 [24]. The plain UHPFRC mixture without CNTs exhibited an average flow value of 220 mm, similar to those of the mixtures with 0.1% and 0.3% CNTs. However, the mixture with 0.5% CNTs had a noticeably lower flow value of 180 mm, although approximately 50% more SP was incorporated, due to insufficient dispersion of the high volume of CNTs. This is consistent with the findings from Kang et al. [32] that the fluidity of cement paste was reduced by adding CNTs. Therefore, samples composed of the mixture with 0.5% CNTs were slightly vibrated to allow them to effectively fill the molds.

**Figure 7:**
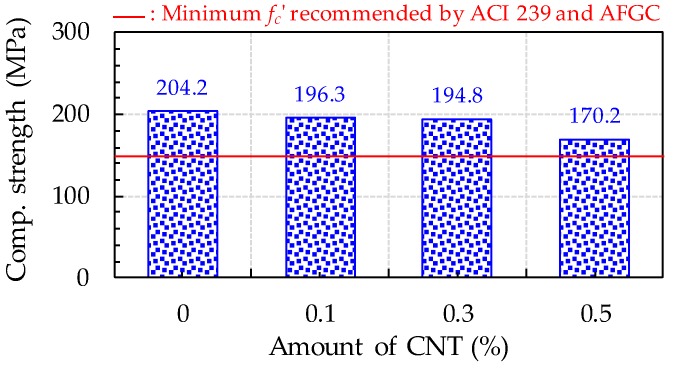
Effect of CNT amount on compressive strength of UHPFRC (Note: *f_c_*’, compressive strength).

To evaluate the implications of CNT content on the compressive strength of UHPFRC, three cubic specimens of dimensions 50 × 50 × 50 mm^3^ were fabricated and tested for each variable. Based on a previous study performance by Han et al. [33], the UHPFRC cubic specimens without the copper plate were used to measure their compressive strength according to the amount of CNTs because the copper plates inserted in the cubes can decrease their strength due to a poor bonding property. As shown in Figure 7, the compressive strength of UHPFRC was reduced by including the CNTs and affected by the amount of CNTs included. The highest compressive strength was found to be 204 MPa for the plain UHPFRC without CNTs, approximately 4% higher than those of UHPFRCs with 0.1% and 0.3% CNTs and 20% higher than that of UHPFRC with 0.5% CNTs. Thus, up to the CNT volume fraction of 0.3%, even though CNT inclusion decreased the compressive strength of UHPFRC, the magnitude of this strength decrease was quite minor, only 4%. However, when the high volume fraction of CNTs, 0.5%, was used, noticeably lower compressive strength was obtained due to their insufficient dispersion. This is inconsistent with findings from previous studies [10,34] for ordinary cement pastes, in which the addition of CNTs generally improved the compressive strength of cement paste. The reason that adding CNTs yielded similar or lower compressive strength of UHPFRC was that the detrimental effect of CNTs, such as insufficient dispersion, more greatly affected compressive strength than their positive effect of inhibiting microcrack propagation. Because UHPFRC exhibits very high compressive strength of about 200 MPa based on its very densified microstructures, weak zones formed by bundles of CNTs might more greatly decrease its compressive strength compared to ordinary cement composites. Even though the UHPFRC with 0.5 vol % CNTs had a lower compressive strength of about 173 MPa, this was still higher than the minimum compressive strength (150 MPa) recommended by the ACI committee 239 [35] and higher than AFGC recommendation [36].

**Figure 8:**
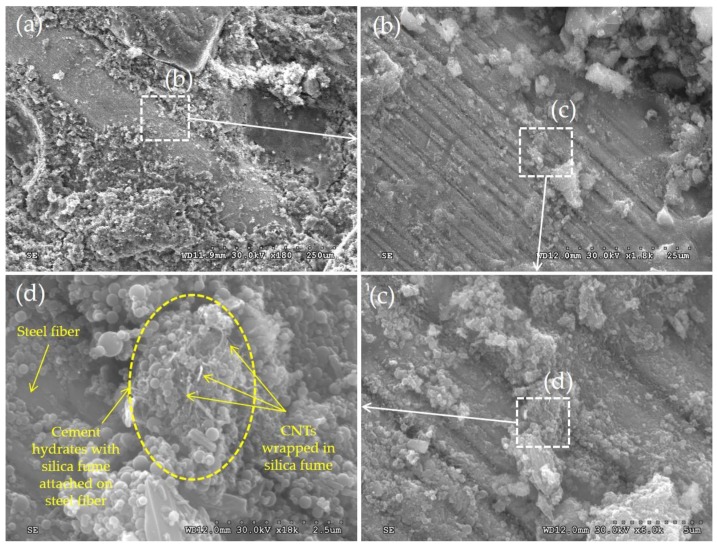
SEM images for UHPFRC with 0.5% CNTs: (**a**) ×180, (**b**) ×1800, (**c**) ×6000; (**d**) ×18,000.

Scanning electron microscopy (SEM) images for UHPFRC with 0.5 vol % CNTs are shown in Figure 8. As listed in Table 3 and Table 4, the size of CNTs was much smaller than that of steel fibers. The surfaces of steel fibers were covered by abundant hydration products and cementitious materials, consistent with findings by Chu et al. [37]. Chu et al. [37] noted that, since the fiber surface can nucleate the deposition of hydration products, mainly calcium hydroxide (CH) crystals and calcium silicate hydrates (C-S-H), approximately 60–70% of the steel fiber surface area was covered by hydration products. Similarly, Chan and Chu [38] reported that, by including 30% and 40% silica fume in reactive powder concrete, abundant cementitious materials adhered to the steel fibers. Based on these observations, it can be inferred that the hydration products were nucleated at the surfaces of steel fibers during the hydration and pozzolanic reaction processes of cementitious materials, namely cement and Zr SF. Due to this nucleation process of hydration products at the surface of steel fibers, lots of CNTs were getting closer to the steel fibers with cement hydrates, as shown in (d) in Figure 8. Once the CNTs are attached to the surface of steel fiber, electrical current can be flowed through the steel fibers. Thus, the conductive pathways were more effectively formed by connections of CNTs and steel fibers, causing a reduced electrical resistivity. For this reason, as was discussed in the Section 3.2.1, the electrical resistivity of UHPFRC without CNTs was substantially reduced by including 0.5 vol % CNTs. However, the resistivity of sonicated UHPFRC reinforced with both 2 vol % steel fibers and 0.5 vol % CNTs was found to be 323 Ω·cm, approximately 2 times higher than that reported for ordinary cement composites reinforced with only 0.5 wt % treated CNTs (called “SPCNT” with a resistivity of about 150 Ω·cm) [39] and 1.0 wt % CNTs (called “MWCNT90” with a resistivity of about 140 Ω·cm) [15], even though a high volume fraction of steel fibers was additionally included. This might be caused by the densified microstructures of UHPFRC, disturbing the connection of CNTs, than other types of cement composites. To precisely explain this observation, however, a further study is required to be done.

**Figure 9:**
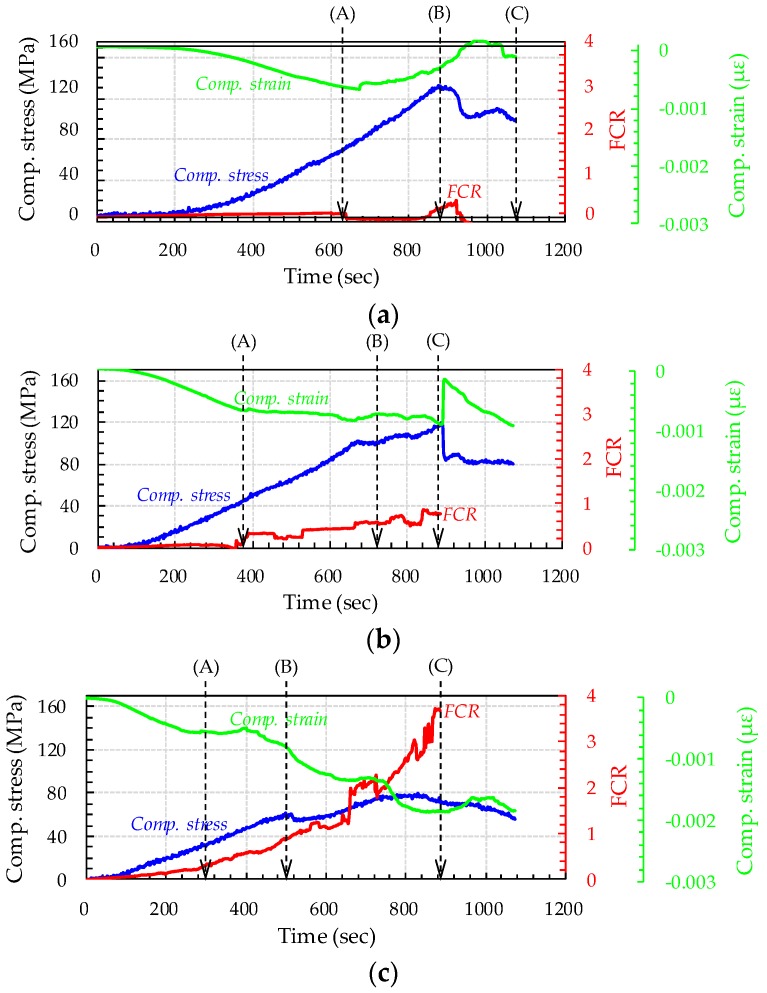
Compressive stress vs. time, strain vs. time, and FCR vs. time curves for UHPFRC with: (**a**) CNT 0.1%; (**b**) CNT 0.3%; and (**c**) CNT 0.5% and capital letters, (A), (B) and (C) are damage process of each specimens.

**Figure 10:**
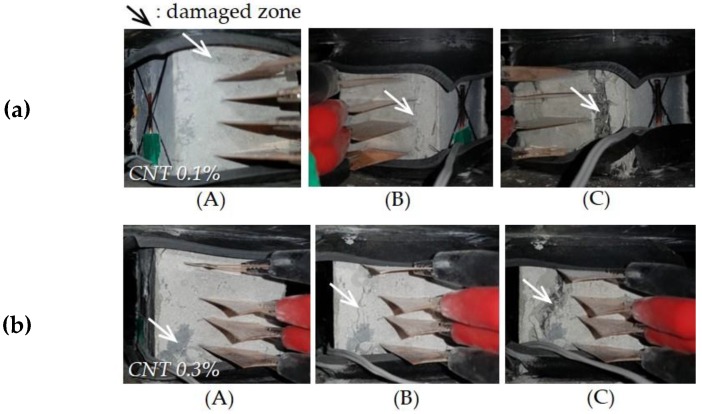

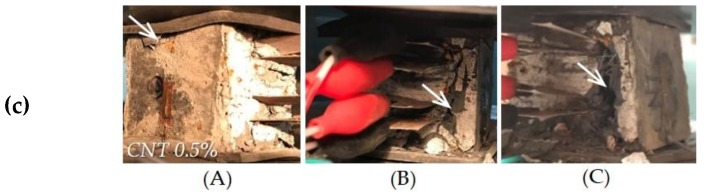
Process of fracture for UHPFRC with CNTs under compression: Small letter (**a**), (**b**) and (**c**) are CNT 0.1%, CNT 0.3% and CNT 0.5%, respectively. Capital letter (A), (B) and (C) indicate damage process of each specimen.

Figure 9 and Figure 10 show curves of compressive stress, strain, and FCR versus time of all cubic specimens, along with their process of fracture. The ultimate compressive stresses in Figure 9 do not indicate the compressive strengths of UHPFRCs with CNTs, since they contained four copper plates, which might decrease their compressive strength [33]. It was obvious that, with increasing CNT content, the FCR increased more drastically under external stress. For instance, the FCR of UHPFRC with 0.5% CNTs at the maximum stress was found to be 2.62, approximately 14.3 and 3.4 times higher than those of UHPFRCs with 0.1% and 0.3% CNTs, respectively. Regardless of the CNT content, the FCR immediately changed, with failure of some portions in the cubic specimens, indicated by the points labeled (A) in Figure 9 and Figure 10. 

**Figure 11:**
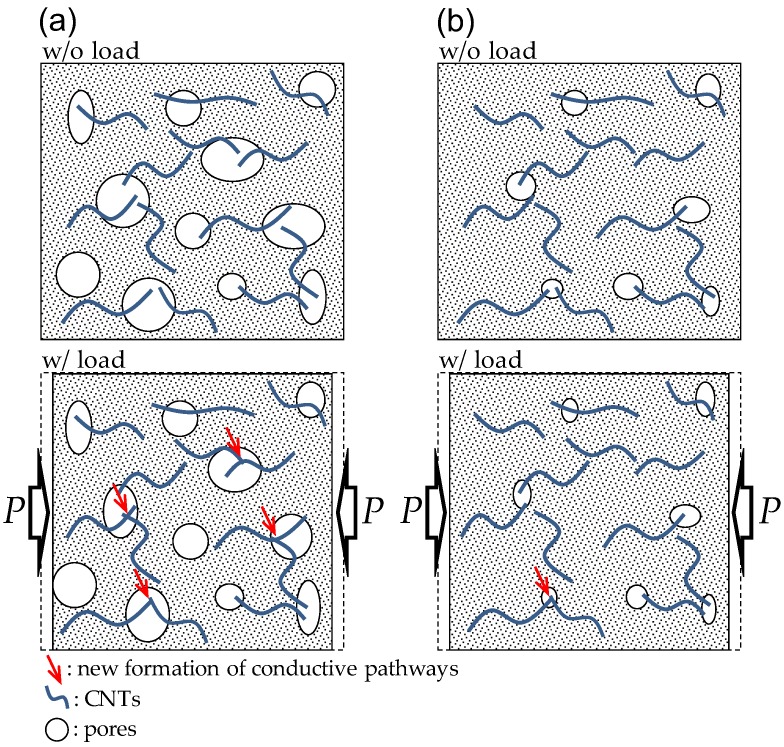
Schematic description of forming new conductive pathways in: (**a**) ordinary cement composites; and (**b**) UHPFRC under compression.

For the UHPFRCs with 0.1% and 0.3% CNTs, the FCR was drastically changed at point (A), while a gradual change in the slope of FCR increase was observed for the UHPFRC with 0.5% CNTs at the point (A). Thus, it can be noted that damage to UHPFRC under compression can be detected by measuring the FCR, regardless of the CNT content. Interestingly, after the formation of cracks (point (A)), the FCR continuously increased for the UHPFRCs with 0.3% and 0.5% CNTs, but changed very little in the case of the UHPFRC with 0.1% CNTs. This suggests that, to evaluate the progressive failure of UHPFRC, the volume fraction of CNT needs to be 0.3% or greater. There was only a minor change in FCR values before point A for all tested specimens, despite nearly linear increases in the compressive stress. This is inconsistent with the findings from previous studies [4,10] that the FCR of cement composites including CNTs obviously changed under a small external compressive stress. This might be caused by much more densified microstructures of UHPFRC compared to ordinary cement composites. As schematically illustrated in Figure 11, the conductive pathways formed by CNT interconnection in UHPFRC were barely changed by the external load due to its very densified microstructures compared with those of ordinary cement composites. The hydration products filling the gaps between CNTs disturb their connection under compressive load, causing insignificant changes in resistivity. 

**Figure 12:**
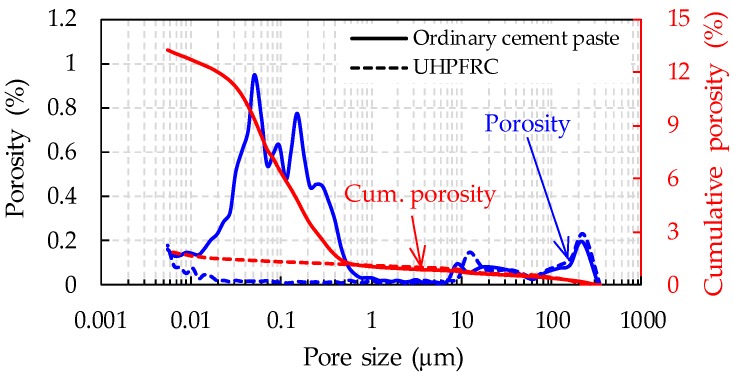
Comparative pore size distribution of ordinary cement paste and UHPFRC.

To support the above explanation, pore size distributions of UHPFRC and ordinary cement pastes with a higher W/B ratio of 0.35 and greater particle sizes were compared based on mercury intrusion porosimetry (MIP) analysis, as shown in Figure 12. It was obvious that most pores smaller than 1 μm that developed in ordinary cement paste were not observed in UHPFRC, leading to much less total porosity. This was mainly caused by the lower W/B ratio of 0.2, the high amount of fine admixtures and their filling effect, and the steam curing with heat applied for UHPFRC. Han et al. [17] similarly reported that using a higher W/B ratio is more effective to improve the piezoresistive performance of CNT/cement composites.

For all cubic specimens, cracks were formed near the inserted copper plates (Figure 10). The direction of cracks formed was perpendicular to the orientation of the copper plates. Subsequently, the cube specimens all failed with widening of the cracks formed near the copper plates (Figure 10). With widening of the cracks, the FCRs of the UHPFRCs with 0.3% and 0.5% CNTs continuously increased, indicating an increase in resistivity. This is because the conductive pathways mainly formed by CNTs and steel fibers were disrupted with increasing crack width.

**Figure 13:**
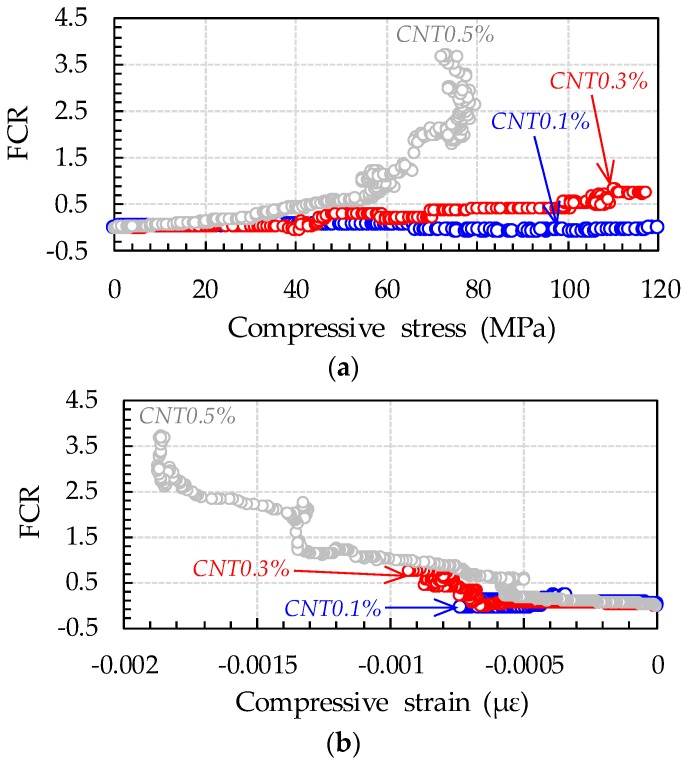
Comparative FCR and: (**a**) compressive stress; and (**b**) compressive strain curves of UHPFRCs with various amounts of CNTs.

Figure 13 compares the trends of compressive stress (and strain) and FCR of UHPFRCs with CNTs under compression. It was obvious that, under small compressive stresses and strains, there was no visible change in FCR values for all tested specimens, whereas the formation of cracks caused the FCR to drastically increase with increasing stress and strain for UHPFRCs with higher amounts of CNTs. Based on these observations, two important conclusions could be drawn: (1) adding CNTs to UHPFRC imparts self-sensing capacity that is activated by the formation of cracks; and (2) increasing CNT content increases the self-sensing sensitivity to compressive stress and strain. Although the ultimate value of compressive stress in Figure 13a did not indicate the compressive strength, since the cubes contained copper plates, the one with 0.5% CNTs exhibited obviously smaller ultimate value of compressive stress than others with smaller amounts of CNTs. This is consistent with the findings from the measurement of compressive strengths mentioned above and possibly due to an insufficient dispersion of CNTs.

#### 3.2.3. Effect of CNT Amount on Flexural Behavior

**Figure 14:**
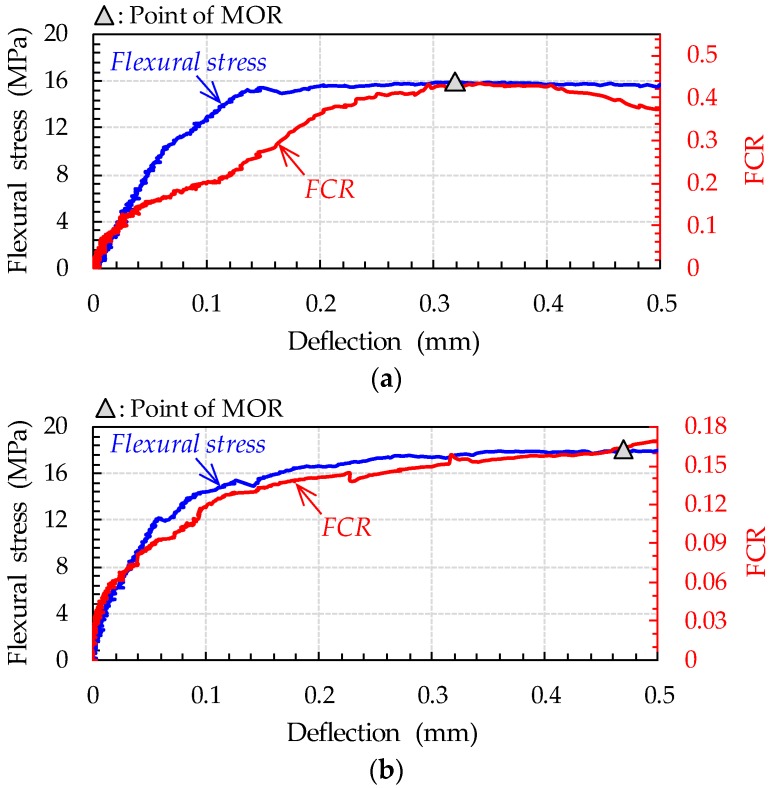
Comparative flexural stress–deflection and FCR–deflection curves for UHPFRC with: (**a**) 0.1% CNTs; and (**b**) 0.3% CNTs.

Based on the compressive test results, to evaluate the progressive failure of UHPFRC, the CNT volume contents need to be equal to or higher than 0.3%. Thus, to verify this observation under different loading conditions, i.e., flexure, the UHPFRC beams with 0.1% and 0.3% CNTs were fabricated and tested according to ASTM C1609 [29]. The comparative flexural stress–deflection and FCR–deflection curves are shown in Figure 14: the flexural stress was calculated by using the following equation, *PL*/*bh*^2^, where *P* is the applied load, *L* is the span length, *b* is the beam width, and *h* is the beam height. For both specimens, the FCR increased with increases in both flexural stress and mid-span deflection. The increase of FCR was mainly caused by the disconnection of conductive materials, such as CNTs and steel fibers, under tensile stress, leading to the increase of electrical resistance. It is interesting to note that the flexural stress was relatively well simulated by the measured FCR before initial cracking as compared with the compressive stress. This indicates that the pre-cracking self-sensing capacity of UHPFRC with CNTs is more effective under tensile stress state than under compressive stress state. Smaller value of FCR was obtained for the UHPFRC including a smaller amount (0.1%) of CNTs than that with a higher amount (0.3%) of CNTs. This uncommon tendency, higher magnitude of FCR for the one with smaller amount of CNTs, is difficult to be explained, since the magnitude of FCR could be influenced by several factors, i.e., number and width of microcracks, fiber orientation, etc. For instance, since more cracks result in more disconnection of CNTs and steel fibers, the number of microcracks, showing great variations, formed in the deflection–hardening region might influence the magnitude of FCR for UHPFRC. Thus, a further study is required for analyzing this observation logically. However, it was obvious that the pre-peak flexural behavior, including elastic region and deflection–hardening region, of UHPFRC was more precisely simulated with a higher amount (0.3%) of CNTs as compared with that having a smaller amount (0.1%) of CNTs, as shown in Figure 14. Accordingly, it is recommended to incorporate 0.3% CNTs into UHPFRC mixture to achieve the self-sensing capacity under flexure.

## 4. Conclusions

This study first examined the effects of steel fiber content, orientation, and pore water content on the electrical and piezoresistive properties of UHPFRC. For this, three steel fiber volume fractions (1%, 2%, and 3%), two casting methods, and two drying conditions (ambient and desiccator drying) were considered. In addition, to investigate the effect of CNT content on the electrical and self-sensing capacities of UHPFRC under both compression and flexure, three volume fractions of CNTs (0.1%, 0.3%, and 0.5%) were additionally incorporated. Based on the above discussions, the following conclusions can be drawn:

(1) Increasing the amounts of steel fibers and pore water in UHPFRC increases its conductivity. The electrical resistivity of UHPFRC was noticeably influenced by the fiber orientation and pore water content, but these effects became insignificant under conditions of higher steel fiber content. Thus, the resistivity of UHPFRC was not noticeably affected by the casting method and pore water content at a fiber volume fraction of 3%.
(2) Including only steel fibers was insufficient to impart piezoresistivity to UHPFRC, although it allowed detection of ultimate failure.
(3) The electrical resistivity of UHPFRC was substantially reduced by including CNTs. Although sonication improved the electrical conductivity of UHPFRC with 0.5 vol % CNTs, the improvement was not sufficient to justify its use.
(4) The compressive strength of UHPFRC decreased by incorporating CNTs. Adding CNTs to UHPFRC imparted a self-sensing capacity that was activated by the formation of cracks, and higher self-sensing capacity was obtained by increasing the CNT content.
(5) The pre-peak flexural behavior, including elastic and deflection–hardening regions, of UHPFRC beams was precisely simulated by measuring the FCR when 0.3% CNTs were incorporated. In addition, the pre-cracking self-sensing capacity of UHPFRC with CNTs was more efficient under tensile stress state than under compressive stress state.

## Figures and Tables

**Table 1:** Mixture proportions.

W/B ^†^	Unit Weight (kg/m^3^)						
	Water	Cement	Zr SF	Silica Sand	Filler (S-SIL10)	SRA	SP *
0.2	175.0	799.5	199.9	879.5	239.9	8.0	18.4

Note: Zr SF, zirconium silica fume; S-SIL10, silica filler with a median particle size of 4.2 μm; SRA, shrinkage reducing admixture; and SP, superplasticizer. * Superplasticizer includes 30% solid and 70% water; ^†^ W/B is calculated by dividing total water contents from mixing water; SP and SRA by total amount of binder.

**Table 2:** Chemical compositions and physical properties of cementitious materials.

Composition % (Mass)	Cement *	Zr SF ^†^
CaO	61.33	0.38
Al_2_O_3_	6.40	0.25
SiO_2_	21.01	96.00
Fe_2_O_3_	3.12	0.12
MgO	3.02	0.10
SO_3_	2.30	-
Specific surface area (cm^2^/g)	3413	200,000
Density (g/cm^3^)	3.15	2.10
Ig. loss (%)	1.40	1.50

* Type 1 Portland cement; ^†^ Zr SF, zirconium silica fume.

**Table 3:** Properties of steel fiber.

Name	*d_f_* (mm)	*L_f_* (mm)	Aspect Ratio (*L_f_*/*d_f_*)	Density (g/cm^3^)	*f_t_* (MPa)	*E_f_* (GPa)
SS fiber	0.20	13.0	65.0	7.9	2788	200

Note: SS fiber, high-strength straight steel fiber; *d_f_*, fiber diameter; *L_f_*, fiber length; *f_t_,* tensile strength of fiber; and *E_f_*, elastic modulus of fiber.

**Table 4:** Properties of CNT.

*d_f_* (nm)	*L_f_* (mm)	*T* (mm)	Layer	Carbon Content (%)	*L_f_*/*d_f_*	*f_t_* (GPa)	*E_f_* (GPa)	Density (g/cm^3^)
15	0.01	3.4–7	-	>90	667	11–63 [27]	270–950 [27]	1.20

Note: CNT, multi-walled carbon nanotube; *d_f_*, diameter; *L_f_*, length; *T*, thickness; *L_f_*/*d_f_*, aspect ratio; *f_t_*, tensile strength; and *E_f_*, elastic modulus.

**Table 5:** Sonication effect on electrical resistivity of UHPFRC with CNTs.

	Sonication Application	Resistivity (Ω·cm)
UHPFRC w/o CNT	X	260.4 × 10^3^
UHPFRC w/0.5% CNTs	X	393
UHPFRC w/0.5% CNTs	O	323

Note: UHPFRC, ultra-high-performance fiber-reinforced concrete; and CNT, multi-walled carbon nanotube.
